# Inflammatory Markers in Patients Using Domiciliary Non-invasive Mechanical Ventilation: C Reactive Protein, Procalcitonin, Neutrophil Lymphocyte Ratio

**DOI:** 10.3389/fpubh.2018.00245

**Published:** 2018-09-05

**Authors:** Birsen Ocakli, Eylem Tuncay, Sinem Gungor, Meltem Sertbas, Nalan Adiguzel, Ilim Irmak, Nezihe Ciftaslan Goksenoglu, Emine Aksoy, Huriye Berk Takir, Ozlem Yazicioglu Mocin, Zuhal Karakurt

**Affiliations:** ^1^Health Sciences University Süreyyapaşa Chest Diseases and Thoracic Surgery Training and Research Hospital, Maltepe, Turkey; ^2^Internal Medicine, Fatih Sultan Mehmet Education and Research Hospital, Ataşehir, Turkey

**Keywords:** domiciliary non-invasive mechanical ventilation, inflammatory markers, CRP, procalcitonin, neutrophil lymphocyte ratio

## Abstract

**Aim:** Early identification and treatment of infections in patients using domiciliary non-invasive mechanical ventilation (NIMV) due to chronic respiratory failure (CRF) can reduce hospital admissions. We assessed C-reactive protein (CRP), procalcitonin, and neutrophil lymphocyte ratio (NLR) as indicators of infection/inflammation.

**Methods:** The study was designed as a retrospective, observational, cross-sectional study, and was performed in 2016 in an intensive care unit outpatient clinic in patients using NIMV. Patients who came to the outpatient clinic with dyspnea, increased sputum, increased prothrombin, and who had hemogram, procalcitonin, and serum CRP, NLR, and PLT/MPV levels assessed, were enrolled into the study. Demographic characteristics, co-morbid diseases, respiratory symptoms, hemogram, biochemistry, CRP, and procalcitonin values in stable and acute attack patients were recorded from patient files. The descriptive statistics and CRP, NLR, and procalcitonin values were assessed.

**Results:** During the study period, 49 patients (24 female) with chronic obstructive pulmonary disease (COPD, *n* = 24), obesity hypoventilation syndrome (OHS, *n* = 15), or interstitial lung disease, *n* = 10), and having had three inflammatory markers assessed, were included in the study. Their mean age was 67 (SD ± 12). Stable patients vs. those who had an acute attack was 41 vs. eight, and within 7 days of outpatient admission four patients were hospitalized. CRP, NLR, and PLT/MPV values were similar in patients' who had sputum purulence, and an increase in dyspnea and sputum, but procalcitonin was significantly higher in patients who had an acute attack. Procalcitonin was not correlated with CRP, NLR, and PLT/MPV.

**Conclusions:** Patients with CRF had similar levels of CRP and NLR during a stable and acute attack state. Procalcitonin may be a better marker for therapeutic decisions in advanced chronic inflammatory diseases.

## Introduction

Long-term oxygen therapy and non-invasive mechanical ventilation (NIMV) are the most appropriate adjunctive treatment options when the underlying disease does not improve despite optimal medical treatment in chronic hypercapnic and hypoxemic respiratory failure (1). The majority of these patients have advanced obstructive and restrictive respiratory function. Timely differentiation of the etiology of respiratory deterioration is crucial for preventing morbidity and mortality in these patients with chronic respiratory failure.

Early diagnosis and treatment of respiratory infections in patients with chronic respiratory failure is crucial for control of the disease in response to treatment. In patients with chronic respiratory failure, symptoms such as dyspnea, and increased sputum amount and purulence help physicians to diagnose pulmonary infections promptly. Increased leukocytes and increased inflammatory markers support the diagnosis of infection.

C reactive protein (CRP) is a well-known inflammatory marker and has been compared with procalcitonin in various studies (2–5). Recently, the neutrophil/lymphocyte ratio (NLR) has been used to diagnose both inflammatory and infectious conditions, and for follow-up treatment outcomes (6–8). Previous studies showed that NLR values have altered to normal values during the course of treatment parallel with the patient's clinical response (9). CRP and procalcitonin are not as cheap or easy to obtain in an emergency unit setting.

CRP values increase with the severity of the infection but do not provide further information on short or long-term outcome. Procalcitonin levels at presentation do not reflect the severity of infection but may provide valuable data during the course of infection, as well as treatment outcomes following biotherapy (10–13).

Studies on the utility of NLR, an easy, fast, and cheap marker for early diagnosis, treatment and follow-up of infection in chronic respiratory failure patients, are limited. In our study, NLR values were assessed in patients with chronic respiratory failure who were using domiciliary NIMV to investigate whether NLR is as valuable as CRP and procalcitonin for the suspicion of infection and assessment of treatment response.

## Methods

The was an observational, retrospective, cross sectional study conducted in a tertiary government teaching hospital for chest diseases and thoracic surgery. The study was conducted from the 1st June 2016 to the 31st January 2017. The study was approved by the local ethics committee of the institution and was conducted in accordance with the ethical principles stated in the Declaration of Helsinki. Due to the retrospective nature of the study, signed informed consents were not obtained.

### Patients

Patients with previously prescribed long-term oxygen therapy and domiciliary NIMV due to chronic respiratory failure due to underlying diseases (chronic obstructive pulmonary disease [COPD]; obesity hypoventilation syndrome [OHS], or lung parenchymal diseases [bronchiectasis or interstitial lung diseases]), admitted to the respiratory intensive care out-patient clinic, and who had CRP, NLR, and procalcitonin levels recorded, were included into the study.

### Reasons for domiciliary NIMV

Previous long-term, home NIMV and oxygen therapy prescriptions was given according to international and national long-term domiciliary NIMV indications (Social Security Institution-Health Practice Statement- SUT 2008 version 2013).

### Definitions

Chronic hypercapnic and hypoxemic respiratory failure was defined by arterial blood gas (ABG) values if patients were in a clinically stable state, had a pH above 7.45 and partial arterial carbon dioxide (PaCO_2_) pressure above 45 mmHg, and partial arterial oxygen pressure (PaO2) below 55 mmHg (1, 14). Obstructive lung disease was defined according to spirometry values if forced expiratory volume in 1 s over forced vital capacity was equal or less than 70%, and restrictive lung disease was defined if greater than 70% (15). An acute exacerbation of chronic respiratory failure due to COPD, OHS, and parenchymal lung was determined using Anthonisen' criteria, namely increasing dyspnea together with increasing sputum volume and purulence, and pulmonary specialist written notes for the presence of an acute attack (16).

### Calculations and measurements

Neutrophil to lymphocyte ratio (NLR) was calculated as neutrophil absolute value/lymphocyte absolute value and platelet to mean platelet volume (PLT/MPV) was calculated as platelet count/mean platelet volume. An NLR value as an indicator of an infectious COPD exacerbation was accepted as ≥3.54 (17). ABGs were done on the day of the outpatient clinic routine visit; ABG's (Rapidlab, Bayer, Leverkusen, Germany) were analyzed at rest from the radial artery. Blood samples were taken during spontaneous breathing of room air, if possible, or during the subject's usual oxygen flow. Spirometry (Zan GPI 3.00, Spire Health, Longmont, CO, USA) was recorded over the previous 12 months and was performed according to the American Thoracic Society guidelines by our center spirometry laboratory (18).

Serum CRP was measured using the nephelometry method with a BN II System (Siemens, Munich, Germany).The normal range of CRP is 0–5 mg/L.

Procalcitonin was measured at a secondary biochemistry laboratory using lateral chromatography and immune turbidimetry, as reported in other studies (19). Procalcitonin levels were accepted as “negative” if < 0.10, “weak positive” if equal or greater than 0.1 and < 0.50, “moderate positive” if equal or greater than 0.5 and < 2.0, and “strong positive” if > 2.0 (20, 21).

### Data recording

The reasons for chronic respiratory failure, underlying co-morbid diseases, demographic characteristics, the previous year's spirometry test results, arterial blood gas values, and hemogram values including neutrophil, lymphocyte, eosinophil, mean platelet volume (MPV), CRP, and procalcitonin values on the day of admission to the outpatient clinic, were recorded from the patient's files. Presence of increased dyspnea, increased sputum production, sputum volume, and color change, and sputum purulence were recorded with physician notes for the diagnosis of acute exacerbation of chronic respiratory failure.

### Statistical analysis

This was designed as an observational, retrospective, cross-sectional study. The portable SPSS package (Version 20, SPSS, Chicago, IL) was used for analysis. Patient groups according to the presence or absence of acute exacerbation were compared using the student *t*-test if continuous values were normally distributed, and the values were defined as the mean ± standard deviation (SD). The Mann Whitney U test was used if continuous values were non-normally distributed and values were defined as the median and 25–75% as interquartile ratio (IQR). Dichotomous variables were compared using the Chi Square test. The Spearman Rank correlation test was used for inflammatory markers. *P* was accepted to be significant if <0.05.

### Results

During the study period, 49 eligible outpatients (24 with COPD, 15 with OHS, and 10 with parenchymal lung disease) were included in the study. According to the patients' symptoms and clinical data, eight (16%) were experiencing an acute attack. All patient hospital visits were routine out-patient visits. Table 1 shows a comparison of stable chronic respiratory failure (CRF) patients versus those CRF patients experiencing an acute attack. Their demographics were nearly similar, but those patients in the acute phase had significantly lower oxygen saturation (*p* = 0.040) and higher pH values (*p* = 0.008) (Table 1).

**Table 1:** Chronic respiratory failure patient demographics and co-morbidities.

**Patients**	**Stable, *n* = 41**	**Attack, *n* = 8**	***p*-values**
Gender, male (%)	20 (49)	5 (63)	0.48
Age, year	67 ± 11	65 ± 15	0.70
Body mass index kg/m^2^	31 ± 10	30 ± 10	0.67
**SMOKING HISTORY *N* (%)**			
Non-smoker	21 (51)	3 (38)	0.53
current smoker	8 (20)	3 (38)	
Ex-smoker	12 (29)	2 (25)	
**CO-MORBID DISEASES**			
COPD	20 (49)	4 (50)	0.95
Diabetes mellitus	10 (24)	3 (38)	0.44
Hypertension	19 (46)	3 (50)	0.65
Congestive heart failure	11 (27)	4 (50)	0.19
**REASONS FOR CRF**, ***N*** **(%)**			
COPD	20 (49)	4 (50)	0.80
OHS	12 (29)	3 (37)	
Brochiectasis/ILD	9 (22)	1 (13)	
**PULMONARY FUNCTION TESTS**			
Obstructive, *n* (%)	22 (54)	5 (63)	0.65
Restrictive, *n* (%)	19 (46)	3 (38)	
**SPIROMETRY TEST**			
FEV1, mL	873 ± 400	858 ± 352	0.92
FEV1, % predicted	39 ± 16	35 ± 11	0.46
FVC, mL	1,278 ± 707	1,585 ± 881	0.29
FVC, % predicted	44 ± 16	44 ± 14	0.68
FEV1/FVC	69 ± 13	70 ± 14	0.87
**ARTERIAL BLOOD GASES**			
pH	7.396 ± 0.029	7.434 ± 0.029	0.008
PaCO_2_, mmHg	47.9 ± 6.6	48 ± 4.1	0.99
PaO_2_/FiO_2_	345 ± 80	313 ± 89	0.36
Sat O_2_, %	93 ± 5	90 ± 5	0.040
HCO3, mmol	28.7 ± 3.9	31.6 ± 2.9	0.057

*COPD, chronic obstructive lung disease; OHS, obesity hypoventilation syndrome; ILD, interstitial lung disease; FEV1, forced expiratory volume in 1 s; FVC, forced vital capacity; PaCO_2_, partial arterial carbon-dioxide pressure; PaO_2_/FiO_2_, partial arterial oxygen pressure over inspired fractionated oxygen ratio; HCO3, bicarbonate*.

Table 2 shows a comparison of inflammatory markers in stable CRF patients versus those in an acute attack state. Procalcitonin levels were significantly higher in patients in the acute state (*p* = 0.001) (Table 2). Leucocyte, NLR, PLT/MPV, and CRP levels were similar in both groups. Of the 49 patients, one had negative procalcitonin levels, one had moderate levels, and 47 showed weakly positive levels.

**Table 2:** Infection and inflammatory markers in patients with chronic respiratory diseases using long-term, non-invasive mechanical ventilation.

	**Stable, *n* = 41**	**Attack, *n* = 8**	***P*-values**
White blood cells × 10^3^	8.50 (6.90–9.60)	9.35 (6.88–10.75)	0.55
NLR	2.88 (2.36–3.91)	3.14 (2.26–4.76)	0.72
PLT/MPV × 10^3^	29.9 (22.5–37.1)	26.6 (21.9–35.2)	0.62
CRP	19.2 (8.8–27.2)	23.1 (5.1–32.1)	0.76
Procalcitonin	0.10 (0.10–0.12)	0.22 (0.183–0.361)	0.001

*Non-parametric Mann Whitney U-test, median (25–75%). NLR, neutrophil to lymphocyte ratio; PLT/MPV, platelet/mean platelet volume; CRP, C-reactive protein*.

Procalcitonin levels were compared with leucocyte, NLR, CRP, and PLT/MPV values (summarized in Table 3) and were found to be not well correlated with these anti- inflammatory markers.

**Table 3:** Correlation of procalcitonin and other inflammatory markers in chronic respiratory failure patients.

**Inflammatory markers**	***r* values**	***p*-values**
Neutrophil to lymphocyte ratio	0.13	0.38
C- reactive protein	0.20	0.18
Platelet to mean platelet ratio	−0.04	0.77
Leucocyte	0.19	0.09

*Analysis by Spearman's Rho correlation*.

Table 4 shows the number of patients with CRF using domiciliary long-term NIMV relative to the parameters used to identify an infectious clinical state. Table 4 also shows the number of patients hospitalized within 7 days of admission to the outpatient clinic.

**Table 4:** The number of patients with CRF using domiciliary long- term NIMV relative to the 4 parameters to identify an infectious clinical state and hospitalized patients in 7 days after the admission to the outpatient clinic.

	**Anthonisen**						NLR^*^		**CRP#**		**Procalcitonin**			
	**Dsypnea**+		**Sputum**+		**Purulance**+		**<3.54**	**≥3.54**	**<5 mg/dL**	**≥5 mg/dL**	**Negative**	**Low +**	**Intermediate +**	**High+**
	**Yes**	**No**	**Yes**	**No**	**Yes**	**No**								
Patient (N)	19	30	15	34	9	40	33	16	8	41	1	47	1	–
Hospitalization	3	1	3	1	3	1	1	3	1	3	–	3	1	–

* Tuncay et al. (17), # C reactive protein, normal level below 5 mg/dL; α, in first week hospitalization.

*CRF, Chronic Respiratory Failure; NIMV, Non-invasive mechanical ventilation; NLR, Neuthrophil to lymphocyte ratio*.

Among the eight patients in the acute attack state, three were hospitalized within 7 days. Seventy-five percent of these patients had positive Anthonisen's criteria, higher CRP and higher NLR levels (Table 4). Procalcitonin levels were weakly positive in all the CRF patients except one who had moderately positive levels of procalcitonin.

## Discussion

This study shows the behavior of inflammatory and infection markers in patients with chronic respiratory failure using long term NIMV due to COPD, OHS, interstitial lung disease, and bronchiectasis. All patients had weakly positive procalcitonin levels. Only 16% of the patients assessed had evidence of an acute clinical attack without requirement for hospitalization in the follow-up. Procalcitonin values were not well correlated with NLR, CRP, and PLT/MPV in patients with chronic respiratory failure.

Procalcitonin and CRP values are used in patients with acute exacerbation of COPD (AECOPD) to attain a quick indication of the etiology in order to initiate antimicrobial treatment as early as possible (22). Very recently Gao and colleagues studied 20 patients with AECOPD and their levels of procalcitonin and high-sensitivity CRP (hs-CRP) (23). They found that serum procalcitonin levels and hs-CRP were well correlated to each other and that they could be used as an auxiliary indicator in the diagnosis of AECOPD, and in assisting the physician in choosing the appropriate antimicrobial therapy (23). A further study by Colak and co-workers compared the usefulness of CRP and procalcitonin in community acquired pneumonia and AECOPD (24). They claimed that serum CRP could be used instead of procalcitonin to diagnose lower respiratory tract infections and determine whether they needed antibiotics or not (24). In contrast, Tanriverdi and colleagues showed that, compared with CRP and NLR, procalcitonin levels were a better indicator of bacterial versus non-bacterial exacerbation in COPD patients (*n* = 77) (25). They also showed that the levels of procalcitonin, CRP, and NLR were above 0.40, 91.5, and 11.5 mg/L respectively (25). In the present study, nearly half of the patients had COPD and the procalcitonin, CRP, and NLR values were lower than those reported in Tanriverdi's study. This may be due the fact that our samples were taken during a routine outpatient clinic follow-up.

Over the last a few years there have been a number of studies assessing NLR as an infectious marker. One study showed that for patients in the emergency unit NLR can be used as a simple, and even better, infection marker for predicting bacteremia than leucocytes and CRP (26).

Jager and co-workers retrospectively evaluated adult patients with suspected community- acquired bacteremia (CAB) in the emergency department. They included blood culture positive (*n* = 92) and blood culture negative (but with suspected CAB) (*n* = 92) patients and compared CRP and NLR values (26). They showed that the NLR together with lymphocytopenia are better predictors for bacteremia than CRP (26). Another study by Jager and coworkers prospectively assessed the prognostic value of NLR in patients admitted to the emergency unit with community acquired pneumonia (27). They reported that NLR had higher prognostic accuracy as compared with traditional infection markers such as CRP neutrophil count and white blood cell (WBC) count.

Very recently, Forget and co-workers defined normal NLR values in a healthy, non-geriatric, adult population to be between 0.78 and 3.53 (28). In the present study on patients with chronic respiratory failure the NLR values were a bit higher than these normal levels.

### Limitations

There were some limitations of this study. First, it was a retrospective, single center study, and second, the study population was small and specific for chronic respiratory diseases with COPD, OHS, and ILD, thus the results were not generalized for the whole population. However, the results presented here may assist physicians when deciding whether the patient has an infectious or chronic inflammation. Assessing the inflammatory and infectious markers can often be confusing for physicians when diagnosing an infection and deciding on antibiotic treatment.

## Conclusions

Patients with chronic respiratory failure using domiciliary NIMV and LTOT with underlying COPD, OHS, or interstitial lung disease, and who have severe dyspnea and sputum, may not easily recognize the progression of their condition. In the follow-up period, an NLR equal or greater than 3.54 may be a better test than CRP (5 mg/dL and above) for recognizing an infection and need for hospitalization. In the present study the majority of patients had weakly positive procalcitonin levels; patients with chronic disease states have pre-dominantly low levels of procalcitonin. Low levels of procalcitonin do not help the physician when making decisions on antibiotic treatment or hospitalization. However, CRF patients with intermediate procalcitonin levels should be followed closely with antibiotic treatment and potentially hospitalized. When differentiating an infection from chronic inflammation in patients with chronic respiratory failure NLR is easy, quick, and cheap to perform, CRP is moderately expensive, and not available in every center, and procalcitonin is expensive and also not available in every center. They should be measured in a clinically stable state at least once for basal levels in patients with CRF. During the follow-up period any changes in procalcitonin, NLR, and CRP values can help the physician to make treatment choices. In clinical practice NLR can be used in place of CRP and procalcitonin.

## Author contributions

BO: contributed to conception and design, acquisition, analysis, interpretation of data, drafting the submitted article, and provided final version. ET: contributed to conception, interpretation of data, critical revision of the submitted article for important intellectual content, and provided final approval of the version. SG: contributed to conception, design, critical revision of the submitted article for important intellectual content, and provided final approval of the version. MS, NC, and EA: contributed to conception, interpretation of data, and provided final approval of the version. NA and OY: contributed to conception, design, interpretation of data, critical revision of the submitted article for important intellectual content, and provided final approval of the version. II: contributed to analysis, interpretation of data, provided final approval of the version. HB: contributed to conception, design, analysis, critical review of the submitted article for important intellectual content, and provided final approval of the version. ZK: contributed to analysis, interpretation of data, critical revision of the submitted article for important intellectual content, and provided final approval of the version to be published.

### Conflict of interest statement

The authors declare that the research was conducted in the absence of any commercial or financial relationships that could be construed as a potential conflict of interest.
